# Salary of the Atlanta City Doctors

**Published:** 1884-12-20

**Authors:** 


### SALARY OF THE ATLANTA CITY DOCTOR.

We are ashamed to tell what the City Physians, appointed to do the pauper practice of the several wards in the city of Atlanta, receive as a salary for their services ; and yet, as we are curious to hear what other cities do for their physicians, we mention it to see whether any other place can beat us in the matter of economy in the medical treatment of paupers. Perhaps some of our brethren in journalism can tell us of a place where the estimate placed upon professional services is as low ; yet we doubt if such a place can be named. The ward physicians of Atlanta receive the pittance of eighty-five cents a day for their services, and furnish their own medicines !

